# Lung infection or inflammation-a puzzling case of MDA-5 associated juvenile dermatomyositis

**DOI:** 10.1186/s12969-023-00933-5

**Published:** 2023-12-20

**Authors:** Anusha Vuppala, Manush Sondhi, Sarwat Umer

**Affiliations:** 1 Department of Rheumatology, Louisiana State University, Shreveport, LA USA; 2 Department of Internal Medicine, Louisiana State University, 3601 Dee Street, Apt 206, Shreveport, LA 71105 USA

**Keywords:** Dermatomyositis, MDA5, Histoplasmosis

## Abstract

**Background:**

Juvenile dermatomyositis (JDM) is an uncommon inflammatory myopathy predominantly affecting children under 18 years of age. Diagnosis relies on identifying specific clinical features, such as muscle weakness, skin rash, elevated muscle enzymes, and MRI and muscle biopsy findings. Autoantibodies associated with inflammatory myopathy offer valuable prognostic insights and can indicate the risk of internal organ involvement, though they are relatively rare in childhood myopathies. JDM can progress to interstitial lung disease (ILD) if associated with MDA5 antibodies, and immunosuppressive therapy constitutes the primary treatment approach.

**Case presentation:**

We present a unique case of JDM complicated by disseminated histoplasmosis in a 12-year-old African American male cross-country runner with no prior medical history. He presented with unintentional weight loss and a rash on his hands, genitals, and fingertips, which persisted despite previous treatments. Diagnosis of JDM was confirmed through clinical and laboratory evaluations. Over time, the patient developed recurrent fevers, thrombocytopenia, and signs of ILD, leading to the identification of disseminated histoplasmosis as a complicating factor. Appropriate antifungal treatment resolved the infectious condition, while continued immunosuppression aided in managing JDM and ILD.

**Conclusions:**

Juvenile dermatomyositis (JDM) remains a rare and intricate autoimmune disorder affecting young individuals. The presence of MDA5 antibodies in JDM patients can lead to severe complications like ILD, necessitating vigilant monitoring. Management includes immunosuppressive therapy, with glucocorticoids and mycophenolate mofetil proving effective, particularly in Clinically Amyopathic Dermatomyositis (CADM) cases. In cases of refractory disease, intravenous immunoglobulin (IVIG) plays a crucial role, offering a safe and beneficial adjunct to treatment. We emphasize the importance of recognizing atypical presentations of JDM, as it can lead to delays in diagnosis and treatment. Our case highlights the complexities of managing dual lung pathology, where a secondary infection exacerbated lung nodules and thrombocytopenia, while ILD was a consequence of atypical myopathy. Combining antifungal treatment with immunosuppression effectively managed both conditions and follow-up evaluations demonstrated improvement in ILD. Awareness of potential fungal infections in immunosuppressed JDM patients is crucial for successful treatment and patient outcomes.

## Background

Juvenile dermatomyositis (JDM) is a rare form of inflammatory myopathy that primarily affects children under 18 years of age. Its diagnosis relies on identifying specific clinical features such as proximal muscle weakness, skin rash, elevated muscle enzymes, MRI findings, and muscle biopsy results [1]. Inflammatory myopathy patients’ autoantibodies not only offer insights into particular phenotypes but also aid in determining prognosis and the risk of internal organ involvement. These antibodies are particularly scarce in childhood myopathies. If JDM is associated with MDA5 antibodies, it can progress to interstitial lung disease (ILD). The primary treatment approach for JDM involves immunosuppressive therapy.

## Case presentation

**Fig. 1:**
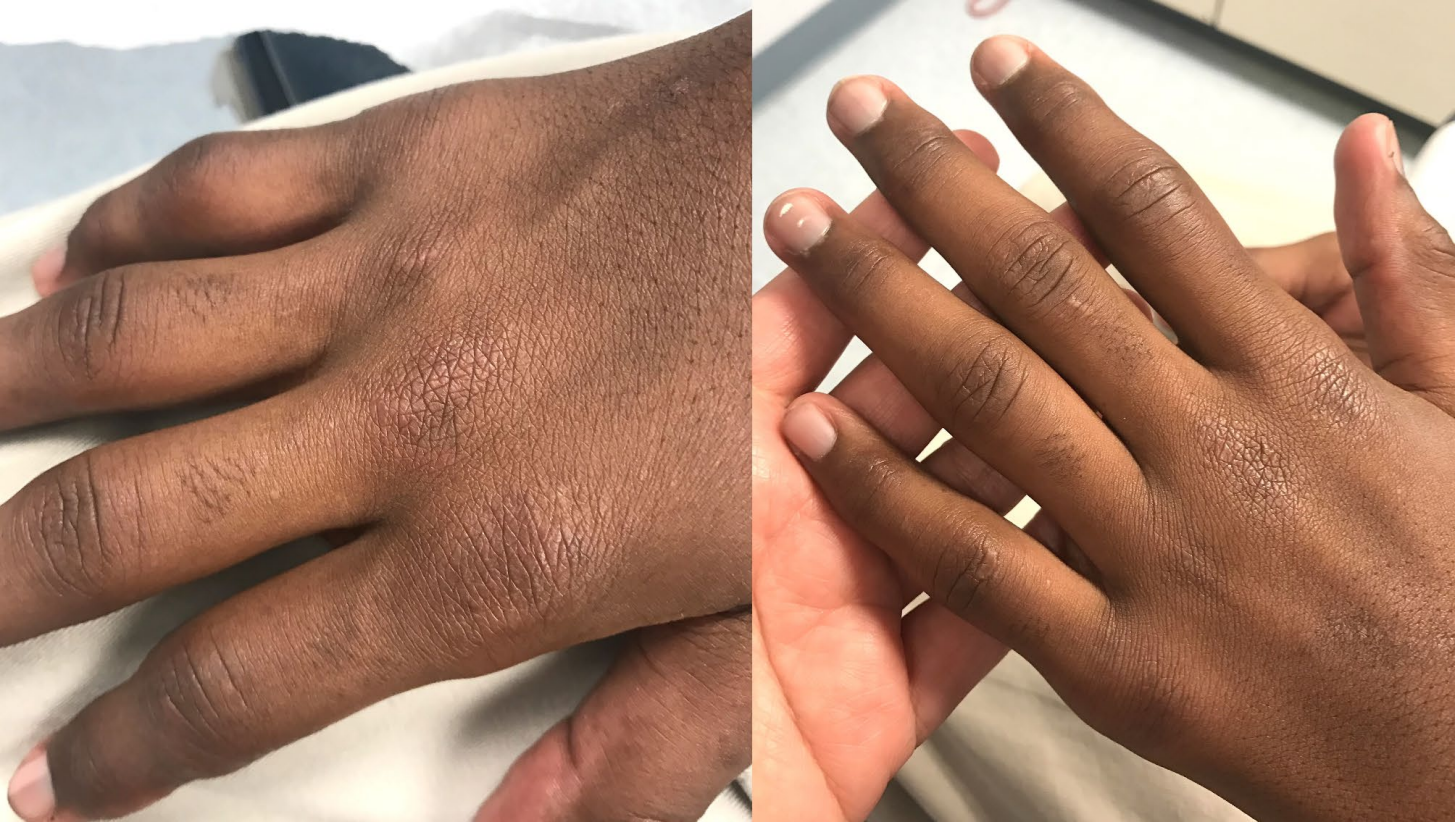
Swelling in the metacarpophalangeal and proximal interphalangeal joints along with the rash

**Fig. 2:**
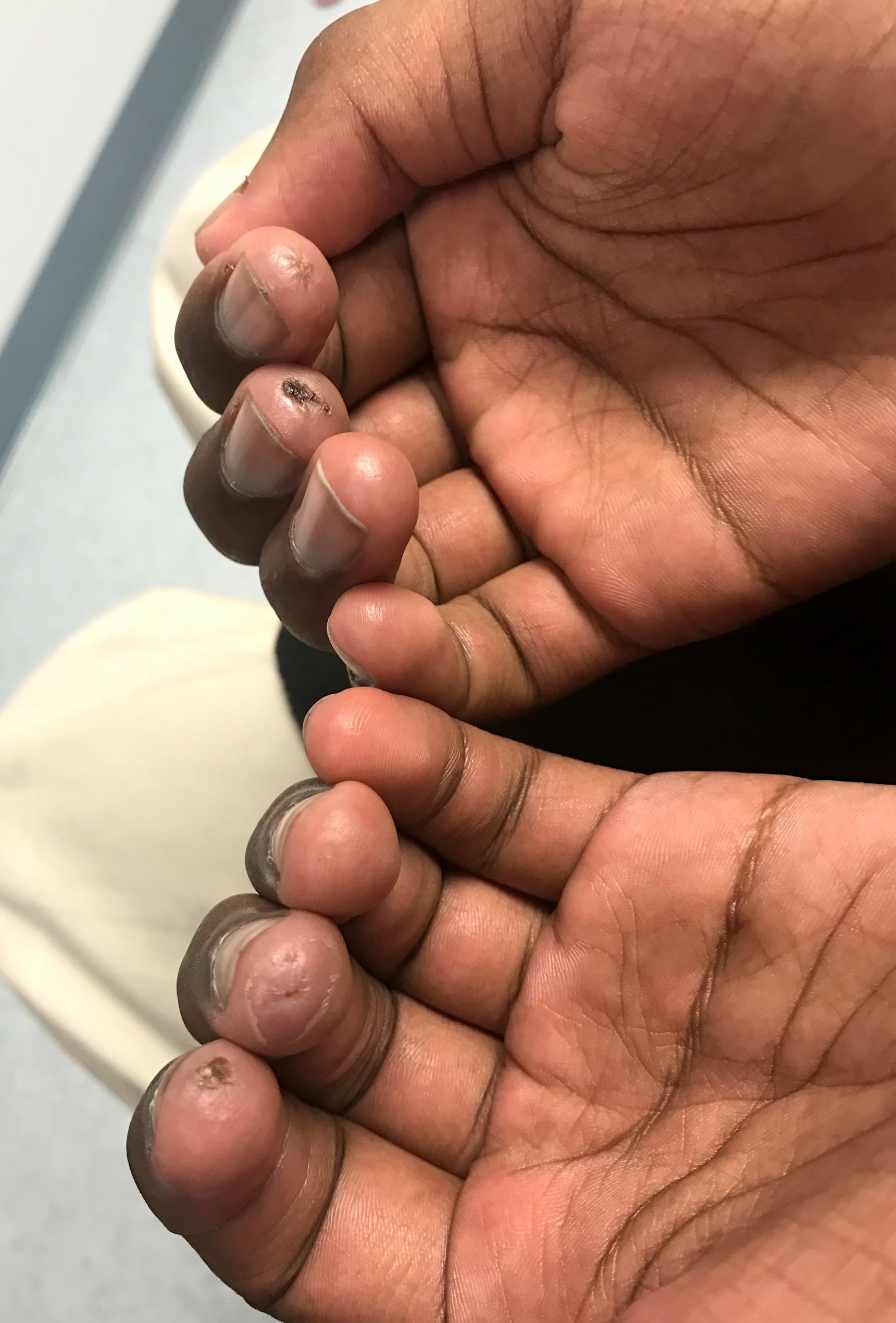
Healed ulcers seen on the tip of the fingers

We present a unique case of JDM complicated by disseminated histoplasmosis. The patient, a 12-year-old African American cross-country runner with no prior medical history, visited the pediatric rheumatology clinic with unintentional weight loss and a rash on his hands, genitals, and blisters on his fingertips. Despite previous treatments, including permethrin, triamcinolone 0.05% topical, and betamethasone 0.05% topical cream, his symptoms persisted. A methylprednisolone dose pack offered temporary relief for the rash. Physical examination revealed swelling in the metacarpophalangeal and proximal interphalangeal (PIP) joints of his fingers, with a blister on the right index fingertip and healed ulcers scattered on the other fingers (Figs. 1 and 2). Lab work showed elevated liver enzymes, lactate dehydrogenase (LDH), and aldolase levels, but normal creatinine kinase (CK) levels. Tests for antinuclear antibody, rheumatoid factor, and cyclic citrullinated peptide antibody were negative. Radiographs of his hands revealed soft tissue swelling around PIP joints. A skin biopsy was consistent with a diagnosis of JDM. Treatment was commenced with prednisone 40 mg daily and oral methotrexate (MTX) 10 mg weekly, along with folic acid 1 mg daily. The MTX dosage was gradually increased to 25 mg weekly, and prednisone was tapered to 10 mg daily over four months.

Several months later, the patient returned to our clinic with new symptoms - recurrent fevers reaching 104 F and a weight loss of 10 pounds. Lab work showed a normal white count, low platelet count of 20,000, mildly elevated liver function tests, LDH, and aldolase, but normal total CK. This atypical presentation included fevers and new-onset thrombocytopenia. Methotrexate was discontinued, and the patient received vancomycin and piperacillin/tazobactam. A chest radiograph revealed infra hilar consolidation and peripheral patchy opacities/nodules. Further examination with computed tomography (CT) chest-abdomen-pelvis showed hepatosplenomegaly, hilar and mediastinal lymph nodes, scattered bilateral pulmonary nodules, and interval development of interstitial infiltrates in both lung bases consistent with new-onset interstitial lung disease (ILD). A pulmonary function test (PFT) was done, which showed a mild restrictive pattern with Forced Vital Capacity (FVC) of 83%, Forced Expiratory Volume in 1 s (FEV1) of 90%, FEV1/FVC of 94%, Total Lung Capacity 80% and Diffusing Capacity for Carbon Monoxide of 75%. Various infectious agents were ruled out through tests, but serum and urine histoplasma antibodies returned significantly high. The patient also underwent a bone marrow biopsy and was found to have disseminated histoplasmosis. Testing for MDA-5 antibodies was also positive due to the new-onset ILD. Histoplasmosis was successfully treated with a two-week course of intravenous amphotericin B followed by oral itraconazole 200 mg twice daily for 90 days (concurrently managed with oral prednisone 5 mg daily). The antifungal treatment led to the resolution of fevers and thrombocytopenia. A follow-up CT scan four months later indicated a resolution of lung nodules and lymphadenopathy but persistent ILD. The patient’s condition is currently stable with a treatment regimen including monthly intravenous immunoglobulin (IVIG), mycophenolic acid, hydroxychloroquine, itraconazole (for prophylaxis), and bactrim (for prophylaxis).

## Discussion and conclusions

Juvenile dermatomyositis (JDM) is a rare and complex autoimmune inflammatory myopathy that primarily affects children under the age of 18. According to the American College of Rheumatology, its incidence is extremely low, with only 1 in 3 million cases reported. Around 10% of patients exhibit myositis-specific antibodies. JDM associated with Anti-MDA5 antibodies often presents with atypical symptoms, including cutaneous ulcerations, oral ulcers, pneumo-mediastinum, erythema or papules over the interphalangeal joints, arthritis, and Raynaud’s phenomenon. It is crucial to closely monitor these patients for lung involvement, as they have a strong association with rapidly progressive interstitial lung disease (ILD) and a poor prognosis. ILD in myositis can be a deadly complication and is often under-recognized, leading to potential delays in diagnosis and treatment [1]. A large multicentric cross-sectional study conducted in Japan revealed that patients with anti-MDA-5 antibodies displayed the lowest survival rates, even when compared to those with malignancy-associated DM [2].

Glucocorticoids remain the cornerstone of therapy for JDM. Additionally, mycophenolate mofetil has shown promising outcomes, especially in cases of Clinically Amyopathic Dermatomyositis (CADM) [3]. In patients with refractory DM involving the lungs and esophagus, intravenous immunoglobulin (IVIG) plays a critical role in management [4]. Studies have demonstrated that IVIG reduces complement activity, membrane attack complex deposition on capillaries and muscle fibers, and the expression of adhesion molecules and cytokine production [5]. The relative safety of IVIG is an important advantage in its use. In our case, a combination of IVIG and mycophenolate mofetil improved ILD and prevented histoplasmosis recurrence. A follow-up CT chest conducted a year later revealed improvement in ILD.

Anti-MDA-5-associated JDM poses diagnostic and management challenges due to its rarity, atypical presentation, and poor prognosis. The presented case is particularly intricate and distinctive because of the dual lung pathology, where lung nodules and thrombocytopenia were a result of coexisting secondary infection, while ILD was a consequence of atypical myopathy. The management was further complicated by concerns of recurrent fungal infection with continued immunosuppression.

## Data Availability

Not applicable.

## Abbreviations

JDM: Juvenile dermatomyositis List of abbreviations
ILD: Interstitial lung disease
CADM: Clinically Amyopathic Dermatomyositis
IVIG: Intravenous immunoglobulin
PIP: Proximal interphalangeal
LDH: Lactate dehydrogenase
CK: Creatinine kinase
MTX: Methotrexate
CT: Computed tomography

## References

[1] Siamak Moghadam-Kia MD (2016). Anti - Melanoma differentiation-Associated gene 5 is Associated with rapidly Progressive lung Disease and poor survival in US patients with amyopathic and Hypomyopathic Dermatomyositis. Arthritis Care and Research.

[2] Chiara Scirocco MD (2020). Rituximab in Antimelanoma differentiation-Associated Protein-5 dermatomyositis with interstitial lung Disease.

[3] Pablo Arturo Olivo Pallo,MD. : Mycophenolate Mofetil in patients with refractory systemic autoimmune myopathies: case series. Advances in Rheumatology; 22;201834. 10.1186/s42358-018-0035-7 30657093

[4] Kazu Hamada-Ode MD. High dose intravenous immunoglobulin therapy for rapidly Progressive interstitial pneumonitis accompanied by anti-melanoma differentiation-associated gene 5 antibody positive amyopathic dermatomyositis. Eur J Rheumatology. 2015;2:83–5. 10.5152/eurjrheum.2015.0076 PMC5047270 27708934

[5] Quick A, Tandan R. Mechanisms of action of intravenous immunoglobulin in inflammatory muscle Disease. Curr Rheumatol Rep. 201113;192–8. 10.1007/s11926-011-0171-0 21503696

